# Stable Fibroblast Growth Factor 2 Dimers with High Pro-Survival and Mitogenic Potential

**DOI:** 10.3390/ijms21114108

**Published:** 2020-06-09

**Authors:** Daria Nawrocka, Mateusz Adam Krzyscik, Łukasz Opaliński, Malgorzata Zakrzewska, Jacek Otlewski

**Affiliations:** 1 Department of Protein Engineering, Faculty of Biotechnology, University of Wroclaw, Joliot-Curie 14a, 50-383 Wroclaw, Poland; daria.nawrocka@uwr.edu.pl (D.N.); mateusz.krzyscik@uwr.edu.pl (M.A.K.); lukasz.opalinski@uwr.edu.pl (Ł.O.); malgorzata.zakrzewska@uwr.edu.pl (M.Z.); 2 Department of Protein Biotechnology, Faculty of Biotechnology, University of Wroclaw, Joliot-Curie 14a, 50-383 Wroclaw, Poland

**Keywords:** FGF2, dimerization, PEGylation, chemical conjugation, regenerative medicine, growth factors

## Abstract

Fibroblast growth factor 2 (FGF2) is a heparin-binding growth factor with broad mitogenic and cell survival activities. Its effector functions are induced upon the formation of 2:2 FGF2:FGFR1 tetrameric complex. To facilitate receptor activation, and therefore, to improve the FGF2 biological properties, we preorganized dimeric ligand by a covalent linkage of two FGF2 molecules. Mutations of the FGF2 WT protein were designed to obtain variants with a single surface-exposed reactive cysteine for the chemical conjugation via maleimide-thiol reaction with bis-functionalized linear PEG linkers. We developed eight FGF2 dimers of defined topology, differing in mutual orientation of individual FGF2 molecules. The engineered proteins remained functional in terms of FGFR downstream signaling activation and were characterized by the increased stability, mitogenic potential and anti-apoptotic activity, as well as induced greater migration responses in normal fibroblasts, as compared to FGF2 monomer. Importantly, biological activity of the dimers was much less dependent on the external heparin administration. Moreover, some dimeric FGF2 variants internalized more efficiently into FGFR overexpressing cancer cells. In summary, in the current work, we showed that preorganization of dimeric FGF2 ligand increased the stability of the growth factor, and therefore, enhanced its biological activity.

## 1. Introduction

Fibroblast growth factor 2 (FGF2) is a member of the family of signaling proteins controlling a plethora of cellular processes, such as proliferation, survival, migration, and differentiation [1,2,3]. FGF2 acts in a paracrine manner by interacting with the four prevalent types of specific transmembrane receptor tyrosine kinases (RTKs), FGF receptors FGFR1–4 [3,4]. FGF2 interacts also with heparan sulfate proteoglycans (HSPG) and this interaction has been shown to regulate its effector functions [5]. The binding of FGF2 together with HSPG to the extracellular part of FGFR evokes receptor dimerization and autophosphorylation of tyrosine residues in the cytoplasmic domain of the receptor, initiating multiple signal transduction pathways. Therefore, FGF2 activity depends on a formation of two 1:1:1 FGF–FGFR–HSPG complexes arranged in a symmetrical dimer [2,6,7]. In view of the fact that FGF2 dimerization is essential for the formation of active ligand–receptor complex, the covalent linkage of two FGF2 molecules should improve the assembly of the FGFR signaling platform. Besides, it was previously reported that many proteins, including FGF2, have a tendency to self-associate to form dimers or higher order oligomers in their native state, since preorganization of multimeric ligands increases the effective local concentrations [8,9]. Hence, dimerization could function as a general mode for sensing ligand concentration, thereby facilitating activation of the receptor. This makes protein dimerization a tempting research tool to create growth factor therapeutics with superagonist activity. 

To this end, Safran et al. dimerized FGF2 through streptavidin–biotin binding and have shown that dimerization reduces affinity to heparin and enhances receptor binding [10]. Accordingly, Kwan et al. demonstrated that genetically engineered dimeric FGF2 construct was characterized by an enhanced biological activity as compared to FGF2 monomer, thereby confirming the importance of FGF2 dimerization [11]. Finally, Decker and coworkers went a step further and searched for optimal linker length for the site-selective conjugation to obtain a covalent FGF2 homodimer with higher biological activity [12].

However, since crystallography and biochemical studies of FGF–FGFR and FGF–FGFR–HSPG complexes have revealed several models of molecular assembly, it was not clear which mode of FGF2 dimerization would be essential for receptor activation [6,7,11,13,14,15,16]. We hypothesized that besides the length of the linker applied for site-specific dimerization, mutual orientation of both FGF2 molecules in a dimer could be even more influential. Here, we present analysis of eight FGF2 dimers of defined topology with diverse biological activities and internalization capacities. We demonstrate that FGF2 dimers are characterized by increased stability, enhanced mitogenic potential and pro-survival activity, as well as induce greater migration responses in cells, as compared to FGF2 monomer. Moreover, various FGF2 dimeric variants exhibit diverse efficiencies of FGFR1-dependent cell uptake. Based on physicochemical properties and biological effects induced by individual dimers, we propose the best candidates for therapeutic applications.

## 2. Results

### 2.1. Design of FGF2 Dimers

**Figure 1:**
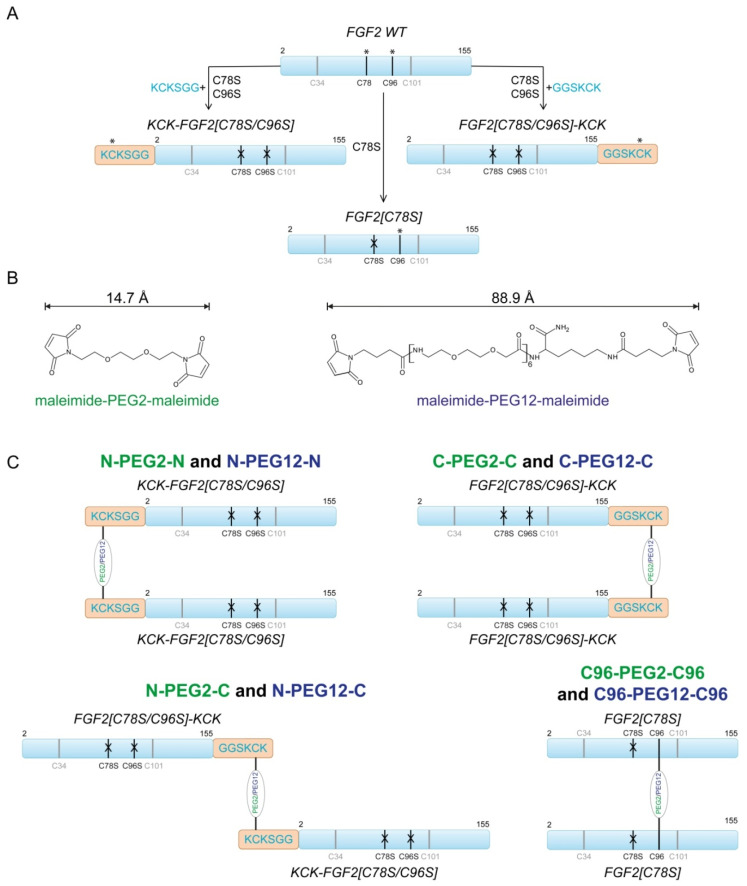
FGF2 covalent dimerization. (**A**) FGF2 variants with maleimide–thiol reaction sites marked with asterisks (*). X defines cysteines mutated to serines. (**B**) Structures of bismaleimido poly(ethylene glycol) linker variants applied for site-specific conjugation. (**C**) Schematic representation of FGF2 dimers’ topology.

The wild-type FGF2 (FGF2 WT) contains two buried and unreactive cysteine residues (C34 and C101) and two exposed and highly reactive ones (C78 and C96) (Figure 1A). However, for the purpose of this work, we used three FGF2 mutants, each containing single surface-exposed reactive cysteine: FGF2[C78S], KCK-FGF2[C78S/C96S] and FGF2[C78S/C96S]-KCK. In the first variant, we substituted C78S surface cysteine with serine. In another two, we mutated both surface-exposed cysteines to serines and introduced N-terminal or C-terminal KCKSGG or GGSKCK extensions, respectively, as we previously found that cysteine surrounded by lysines (KCK sequence) was highly prone to maleimide–thiol reaction (Figure 1A) [17,18].

Application of single cysteine-containing FGF2 mutants allowed for site-specific PEGylation, and therefore, generation of homogenous dimers of defined topology. Moreover, we applied two poly(ethylene glycol) linkers functionalized with maleimide moiety for cysteine conjugation (Figure 1B). We decided to use PEG linkers of two lengths to investigate how the length of PEG linker influences dimers’ biological activity. We hypothesized that long flexible linker could enable protein dimerization without disrupting the mutual arrangement of individual FGF2 molecules in the complex with FGFR. In turn, dimerization via a short linker could force non-native protein conformation, and therefore, affect receptor activation. We choose 90 Å PEG12 variant, which is longer than the inter-cysteine (C96) distance in tetrameric 2:2 FGF2:FGFR1 complex (~80 Å) and, for comparison, short 15 Å PEG2 [6,7,12]. Hereby, we obtained eight FGF2 dimers of different topology and/or linker length. Depending on the linker used for dimerization and localization of cysteine conjugation site, we called them: N-PEG2-N, N-PEG2-C, C-PEG2-C, C96-PEG2-C96, N-PEG12-N, N-PEG12-C, C-PEG12-C, and C96-PEG12-C96 (Figure 1C).

### 2.2. Synthesis of Dimeric FGF2 Variants

**Figure 2:**
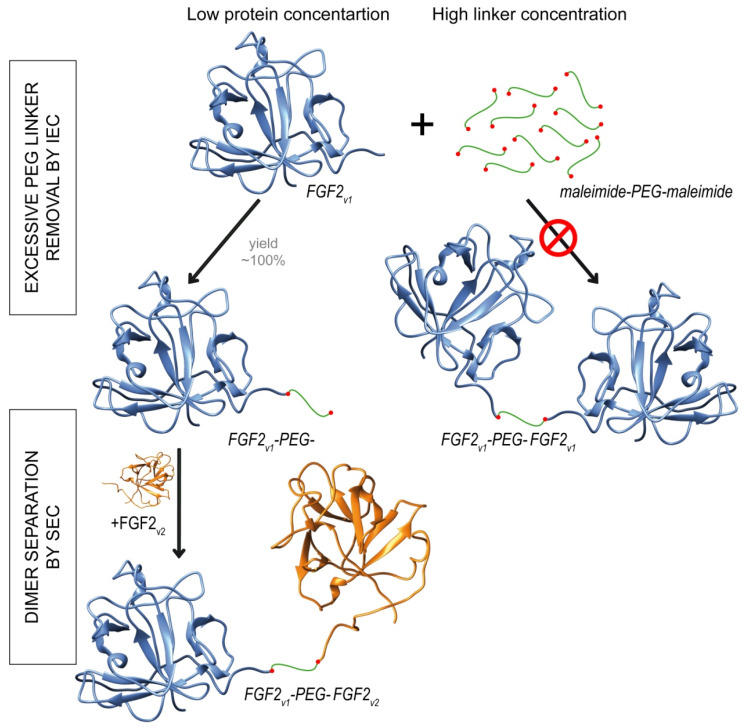
PEGylation at single cysteine residue. Application of low protein concentration (10 µM) and high, 100-fold, molar excess of PEG linker over protein sulfhydryl groups for the reaction, guaranteed full conversion of fibroblast growth factor 2 variant 1 (FGF2_v1_) to PEGylated FGF2_v1_ (FGF2_v1_-PEG-). Excessive PEG linker removal was performed by the means of ion-exchange chromatography and PEGylated protein was subjected to the reaction with excessive concentration of unPEGylated FGF2 variant 2 (FGF2_v2_). Unreacted FGF2_v2_ was removed by size-exclusion chromatography.

**Figure 3:**
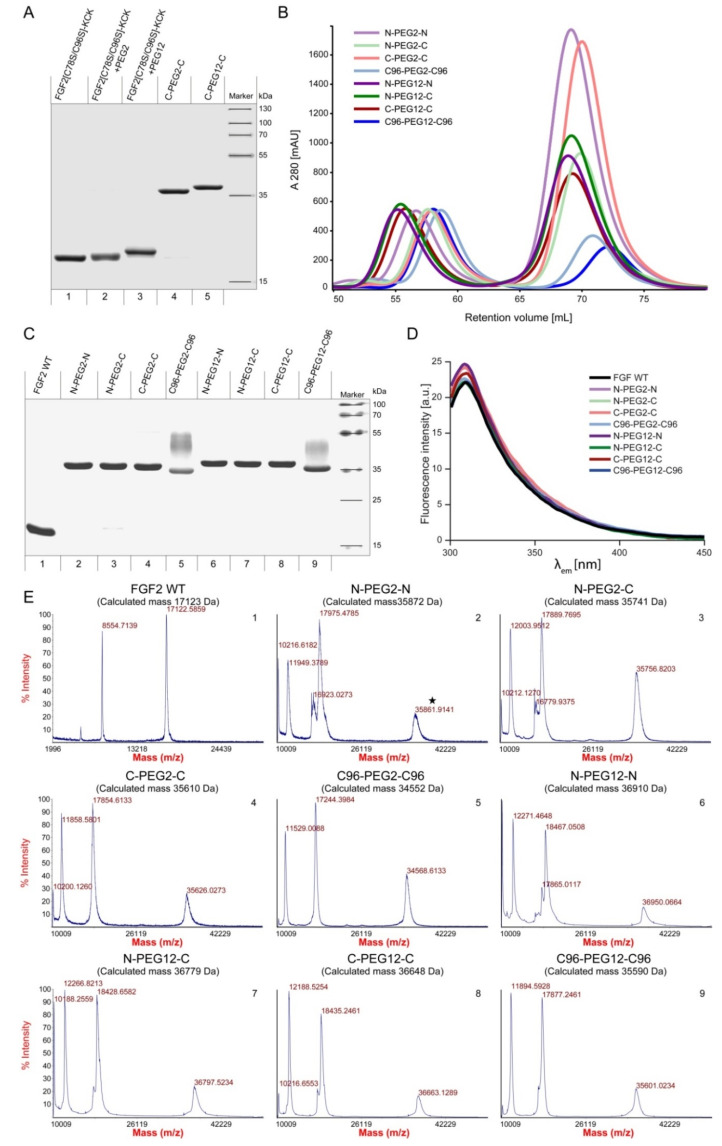
Synthesis, purification and characterization of FGF2 dimers. (**A**) Representative SDS-PAGE evaluation of purified products at individual conjugation reaction stages as shown for C-PEG2-C and C-PEG12-C dimers. Analyses performed for remaining preparations were analogous. (**B**) Size-exclusion chromatography of dimerization reaction mixture. (**C**) Electrophoretic separation of purified FGF2 dimers. (**D**) Fluorescence emission spectra of wild-type FGF2 (FGF2 WT) and dimers. (**E**) Mass spectra of dimeric FGF2 variants. Numbers of MS data correspond to the lane numbers in (**C**).

The schematic representation of dimerization reaction is presented in Figure 2. We produced homogenous FGF2 variants (Figure 3A, line 1) and attached a maleimide-PEG2-maleimide (Figure 3A, line 2) or maleimide-PEG12-maleimide (Figure 3A, line3) linker via maleimide–thiol reaction. 

To ensure the full conversion of FGF2_v_ to FGF2_v_-PEG2 or FGF2_v_-PEG12 in the first step of the reaction, we used low concentrations of protein (10 µM) and high, 100-fold, molar excess of PEG linker over protein –SH groups (Figure 2). Such conditions prevented an uncontrolled FGF2 homodimerization, and therefore, ensured homogeneity of the final product. PEGylated protein was then purified by the ion-exchange chromatography and subjected to the reaction with an excessive concentration of unPEGylated FGF2 variant constituting the second molecule in the dimer. In the last step, unreacted FGF2 monomers were removed using size-exclusion chromatography (Figure 2, Figure 3B). All dimers were homogenous and of high purity, as demonstrated by SDS-PAGE (Figure 3A lines 4 and 5; Figure 3C, lines 2–9). Under reducing/denaturating conditions, C96-PEG2-C96 and C96-PEG12-C96 dimers, unlike the others, are not linear molecules, and therefore, appear as a smear (Figure 3C, lane 5 and 9). The identity of the dimers was confirmed by MALDI-MS (Figure 3E).

To verify the native conformation of FGF2 molecules in the dimers, we employed tryptophan fluorescence measurements. In natively folded FGF2 WT, tryptophan fluorescence is quenched by neighboring histidine and proline residues, and the spectrum is dominated by emission of tyrosine residues (maximum at 303 nm). Upon unfolding, the quenching effect is suppressed, resulting in a significant increase in the fluorescence at 353 nm. The fluorescence emission spectra of all of the dimers were similar to those of native FGF2, showing that FGF2 monomers in all obtained dimers were properly folded (Figure 3D).

### 2.3. Biological Activities of FGF2 Dimer 

**Figure 4:**
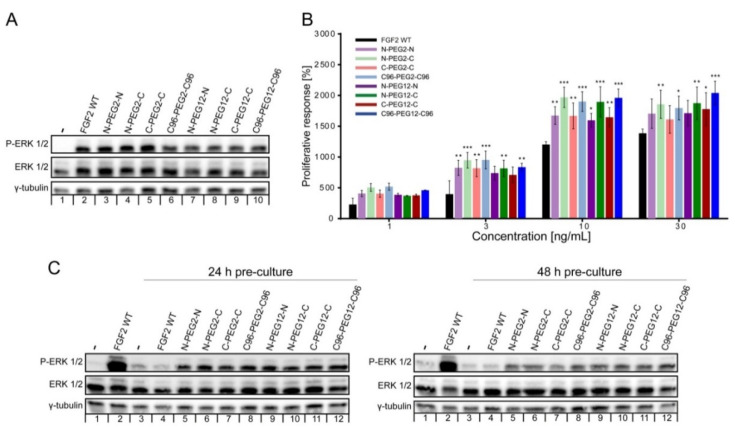
Biological activity and stability of FGF2 dimers. (**A**) Serum-starved NIH3T3 cells were stimulated with 10 ng/mL wild-type FGF2 (FGF2 WT) or dimers for 15 min. Activation of downstream signaling cascades was detected by immunoblotting using the following antibodies: anti-phospho-ERK1/2 (P-ERK1/2), anti-ERK1/2 and anti-γ-tubulin as a loading control. Representative experiment is shown, *n* = 3. (**B**) Serum-starved NIH3T3 cells were treated with FGF2 or dimers at various concentrations (1–30 ng/mL). After 72 h, cell viability was measured using AlamarBlue Reagent. Percent proliferative activity of NIH3T3 (mean ± SD) was normalized to the blank media per treatment set. The average values and errors were calculated based on three independent experiments. Statistical significance: * *p* < 0.05, ** *p* < 0.01, *** *p* < 0.001. (**C**) Stability of dimers upon incubation with NIH3T3 cells. Serum-starved NIH3T3 cells were stimulated for 15 min with either freshly prepared 10 ng/mL FGF2 or cell-conditioned media after 24 h (left panel) or 48 h (right panel) incubation with 10 ng/mL FGF2 or dimers. Activation of FGFR downstream signaling was evaluated by immunoblotting. Representative results from three independent experiments are shown.

To verify whether dimerization did not affect the binding of the FGF2 variants to the cellular pool of FGFRs, we analyzed the activation of FGFR-dependent extracellular signal-regulated kinase ERK1/2 pathway in NIH3T3 fibroblasts and U2OS osteosarcoma cells stably transfected with FGFR1 (U2OS-R1) upon a 15 min treatment with the individual dimers [3]. All dimeric samples stimulated the downstream signaling at the same level as FGF2 WT, as detected by western blotting with anti-phospho-ERK1/2 antibody (Figure 4A and Figure S1). This indicates that all FGF2 dimers are fully competent in the activation of receptor-dependent signaling cascades.

### 2.4. Mitogenic Potential of FGF2 Dimers

Compared to signaling, mitogenic activity of FGFs requires much longer exposure of cells to the growth factor [19,20]. Therefore, the induction of cell division by FGFs is determined by both the ability of growth factors to activate FGFRs and by their long-term stability in the cell culture medium. We applied a standard AlamarBlue viability test to measure the proliferative potential of FGF2 dimers. We found out that all investigated dimers at concentrations from 1 to 30 ng/mL induced significantly greater proliferative response than FGF2 WT (Figure 4B). The highest mitogenic response was evident in cells cultured in the presence of N-C and C96-C96 oriented dimers, regardless of the PEG linker length. The most statistically significant differences were noticed at 10 ng/mL protein concentration, when abovementioned dimers induced over 19-fold increase in the proliferative activity compared to the blank media, while FGF2 WT 12-fold (Figure 4B). The advantage of dimers over FGF2 WT in the context of mitogenic potential was equalized by the addition of heparin (10 U/mL) to the cell culture (Figure S2). Although heparin supplementation increased the mitogenic response of all investigated proteins, the effect was most pronounced for cells grown in the presence of FGF2 WT. At 10 ng/mL, the enhancement in cells’ mitogenic activity caused by FGF2 and heparin supplementation achieved 20.4-fold increase and we did not find any statistically significant differences between all investigated preparations. We hypothesize that in the absence of heparin, FGF2 dimers are more stable in the cell culture medium compared to FGF2 monomer, which stands behind their superior mitogenic potential.

### 2.5. Susceptibility of FGF2 Dimers to Degradation 

To further investigate whether dimers’ stability in cell culture medium influences mitogenic properties, we examined their ability to induce FGFR-dependent signaling upon pre-incubation with cells for 24 and 48 h. We incubated FGF2 WT and the dimers with NIH3T3 cells for 24 and 48 h, then, we collected cell-conditioned media and determined the activation of FGF2-induced signaling cascades with the use of fresh serum-starved NIH3T3 cells [21]. The activation of FGF2-induced signaling pathways was used as a sensitive readout of active growth factor levels in the medium. As shown in Figure 4C there was a tremendous decrease in ERK1/2 phosphorylation level in response to FGF2 WT precultured with cells for 24 h. In turn, the activation of downstream kinases in cells stimulated with dimers was evident, regardless if they had been pre-incubated with cells or not. This indicated that the amount of functional dimers was not significantly altered by the 48 h incubation process, whereas the concentration of active FGF2 WT was strongly reduced already after 24 h of incubation. We assumed that the observed effect is caused either by the denaturation and/or proteolytic degradation of FGF2 WT in the medium. Therefore, dimerization of FGF2 increased the stability of FGF2, and thus, protected against the loss of activity.

### 2.6. Anti-Apoptotic Activity of FGF2 Dimers

**Figure 5:**
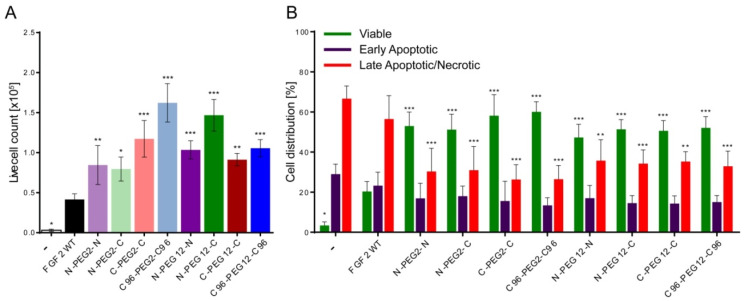
Pro-survival effect of FGF2 dimers on NIH3T3 cells. (**A**) Live cell counting performed with the use of hemocytometer and Trypan Blue staining after 72 h of culture with 10 ng/mL wild-type FGF2 (FGF2 WT) or dimers. (**B**) Apoptosis assessed by Annexin V and propidium iodide (PI) assay. The data presented in panels A and B are mean values of four independent experiments ± SD. Statistical significance (versus FGF2 WT group): * *p* < 0.05, ** *p* < 0.01, *** *p* < 0.001.

Besides the mitogenic properties, FGF2 plays an important role in cell protection against apoptosis [5]. Thus, in the next step, we analyzed the pro-survival effect of FGF2 dimers on NIH3T3 cells. Cell morphology was monitored by light microscopy and the live cell number was determined by Trypan blue exclusion (Figure 5A). After 72 h of culture in the serum-deprived medium with the addition of 10 ng/mL FGF2 WT, we detected 0.4 × 10^5^ live cells. Interestingly, in groups exposed to the same concentration of dimers, the number of viable cells was markedly higher, from 0.8 × 10^5^ to 1.6 × 10^5^. The most abundant were populations subjected to the treatment with C96-PEG2-C96 and N-PEG12-C dimers, in which live cell count was respectively, 3.9 and 3.5-fold higher than for FGF2 WT. Notwithstanding, when we included heparin, cell viability in FGF2 WT-stimulated population increased to the level of dimer-treated ones and we did not observe major discrepancies in the number of live cells in investigated groups anymore (Figure S3A). Moreover, to confirm our results obtained in the cell viability assay and further examine the effect of FGF2 dimers on NIH3T3 fibroblasts, we performed FACS analysis of AnnexinV and propidium iodide staining, allowing us to detect apoptotic and dead cells, respectively (Figure 5B). Analysis of populations of serum-starved cells subjected to the treatment with FGF2 WT for 72 h revealed that most of the cells underwent apoptosis or necrosis, and only 20% of cells remained viable. Consistently with previous data, the viability of cells treated with all dimeric variants was markedly increased—up to 60% in case of C96-PEG2-C96 variant. Addition of heparin partially restored activity of FGF2 WT and resulted in 78% cell viability increase (Figure S3B). Nevertheless, the highest percentage of live cells (~90%) was detected in groups treated with C-PEG2-C, C96-PEG2-C96, N-PEG12-C and C-PEG12-C dimers.

### 2.7. Induction of Cell Migration by FGF2 Variants 

**Figure 6:**
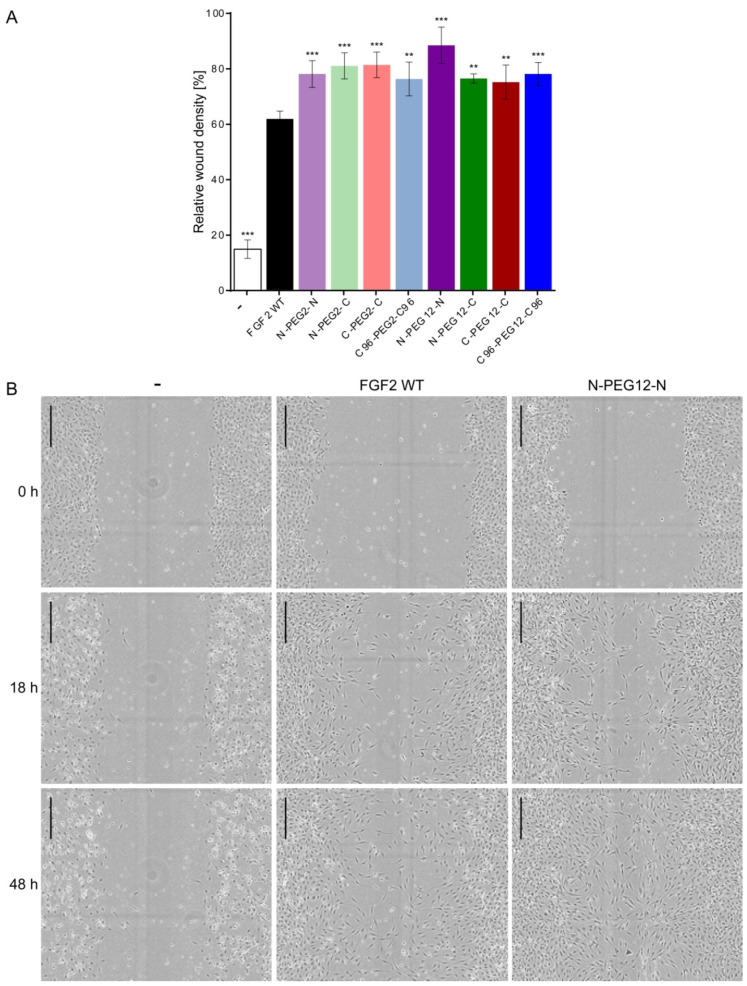
The effect of dimeric FGF2 variants on the migration of NIH3T3 cells. Serum-starved NIH3T3 cells were treated with 10 ng/mL wild-type FGF2 (FGF2 WT) or dimers. (**A**) Relative wound density was calculated after 48 h of cell stimulation. Representative results of two independent experiments are shown. The average values and errors were calculated based on four replicates. The data were obtained by IncuCyte Zoom software and are shown as mean ± SD. Statistical significance (versus FGF2 WT group): ** *p* < 0.01, *** *p* < 0.001. (**B**) Representative images of the wounds at the baseline and 18 h, and 48 h post-scratch, on an example of wild-type FGF2 and N-PEG12-N dimer. Scale bar: 300 µm.

FGF2 is a wide spectrum survival factor, essential regulator of cell division, and also modulator of cell migration [22,23,24]. Therefore, to further assess the functional effect of dimerization we examined the ability of investigated specimens to stimulate cell motility in a standard scratch-wound assay. A constant gap was created in-between the monolayer of NIH3T3 cells, and closure of the “wound” over time was scored, as it is proportional to the migration ability of cells. Graphical data of gap closure rates are given in Figure 6A. FGF2 WT showed significantly reduced potential for induction of cell migration, as compared to dimers. At 48 h post-scratch, wound area analysis revealed 61% confluent cell layer for FGF2-stimulated group, but in groups subjected to the treatment with FGF2 dimers, relative wound density was up to 76–88%. The highest confluency in the scratch region was scored in the group exposed to N-PEG12-N dimer. Representative phase-contrast images of NIH3T3 cells immediately after a scrape injury, 18 and 48 h post-wounding are shown in Figure 6B. Again, the effect of reduced FGF2 ability to induce cell migration could be overcome by the incorporation of heparin. In the presence of heparin, we did not observe significant differences in the response of cells to FGF2 or dimers (Figure S4).

### 2.8. Increased Internalization of Dimeric Variants

**Figure 7:**
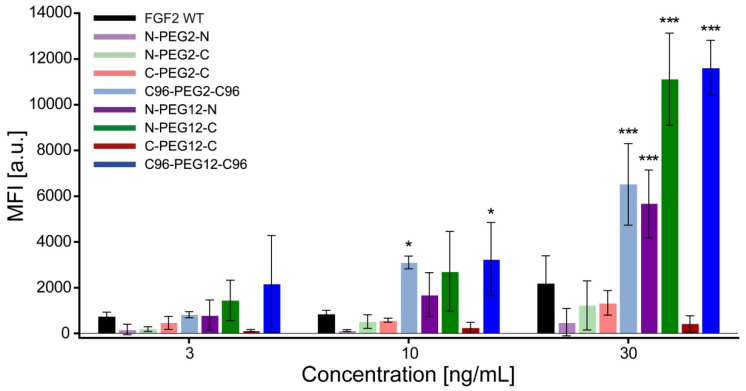
Internalization of fibroblast growth factor 2 (FGF2) dimers analyzed by flow cytometry. For determination of internalization efficiency, U2OS cells stably transfected with FGFR1 (U2OS-R1) were incubated for 20 min at 37 °C with increasing concentrations of Alexa Fluor 488-labeled wild-type FGF2 (FGF2 WT) or dimers (3, 10 and 30 ng/mL). Results represent the mean fluorescence intensities under each experimental condition from three independent experiments. Values of mean fluorescence intensity (MFI) are the means for each data set ± SD. Statistical significance (versus FGF2 WT group): * *p* < 0.05, *** *p* < 0.001.

It is well known that FGF2 can enter the cell via specific FGFR-dependent endocytosis. To check whether its dimeric versions are similarly able to enter the cell, we used a U2OS-R1 model cell line that overproduces FGFR1. We fluorescently labeled FGF2 preparations and determined the efficiency of dimers’ internalization by flow cytometry (Figure 7). In the experimental setup, non-internalized fluorophore-labeled ligand was removed from the cell surface by acid washing. Analysis of the internalization yield versus ligand concentration revealed that C96-PEG2-C96, N-PEG12-N, N-PEG-12-C and C96-PEG12-C96 dimers showed enhanced efficiency of cell uptake. At 30 ng/mL, the increase in mean fluorescence intensity of N-PEG12-C and C96-PEG12-C96 dimers-treated cells was more than fivefold higher than FGF2 (Figure 7). Moreover, the kinetics of internalization of abovementioned variants were shifted towards shorter time points (Figure S5A). To visualize the diverse internalization patterns of FGF2 and dimeric variants, we utilized fluorescence microscopy. Cells were first pre-incubated on ice with DyLight 550-labeled FGF2 WT or dimers to enable binding to cell surface FGFR1, and then, shifted to 37 °C for 15 min to allow for receptor endocytosis. At these conditions, C96-PEG2-C96, N-PEG12-N, N-PEG12-C and C96-PEG12-C96 dimers were efficiently internalized and were visible as numerous spots, likely representing endosomes bearing fluorescently labeled proteins. On the contrary, the majority of DyLight 550-labeled FGF2 WT was retained in large clusters on the cell surface, while only a small fraction was internalized (Figure S5B). 

The lowest internalization yield at all investigated concentrations was observed for C-PEG12-C dimer. To ensure that labeling with fluorescent probe did not interfere C-PEG12-C dimer’s function, we evaluated its capacity to bind FGFR1 using BLI technology (Figure S6A) and confirmed its biological competence in NIH3T3 (Figure S6B) and U2OS-R1 (Figure S6C) cells. The results show that Alexa Fluor 488-labeled C-PEG12-C dimer remained fully competent in the activation of receptor-dependent signaling cascades. Therefore, impaired internalization of this dimer is probably the outcome of unfavorable topology. Together, these results indicate that the arrangement of individual FGF2 molecules in the dimer is pivotal for its ability to enter the cell. 

## 3. Discussion

The focus on tailoring FGF2 research towards medical applications has been undertaken in opposing fields of tissue repair and oncology [4,25,26,27,28,29,30,31,32]. In the current study, we sought to improve FGF2 biological activity for its potential use in the regenerative/repair medical settings, such as treatment of burns, chronic wounds, pressure ulcers, diabetic foot, and critical limb ischemia. Exploiting the available knowledge of FGF2 activity and FGF2 crystal structure in the complex with its receptor, we designed eight topologically different dimeric FGF2 variants to test whether they are more competent in evoking cellular responses. 

Mutations of the FGF2 WT were undertaken in order to obtain a single reactive cysteine for the chemical conjugation, spatially isolated from residues involved in the receptor binding and required for the protein activity. The dimerization was accomplished through the maleimide–thiol reaction with bis-functionalized linear PEG linkers of two lengths. We obtained eight variants of FGF2 dimers, differing in the mutual orientation of FGF2 molecules. We found out that all of the engineered dimers remained functional in terms of FGFR downstream signaling activation and evoking both short-term and long-term cellular responses. We also confirmed that dimeric FGF2 variants did not show any tendency to aggregate and preserved the native conformation, as determined by fluorescence emission spectra that are very sensitive to changes in the tertiary structure [33,34]. Further to this, we showed that the increased stability of the dimers in cell culture medium, in comparison with FGF2 WT, resulted in maintenance of their mitogenic and anti-apoptotic potential after 72 h of culture with NIH3T3 cells.

Decker et al. reported that FGF2 homodimer, conjugated through the surface-exposed cysteine 96 with a long PEG2k linker, induced the greatest activity in HDF cells [12]. In contrast, dimer linked via a small molecule linker, divinyl sulfone, although it did stimulate greater cell growth than FGF2, the increase was very low [12]. The authors hypothesized that this effect was likely due to the steric restrictions of such a short linker. In our studies, we did not observe any differences between the length of PEG linker and the final outcome of the dimer in terms of the mitogenic and pro-survival potential or capacity to induce cell migration. Nonetheless, our data are not directly comparable to this reference since we used a different cell type and Decker et al. added FGF2 proteins to EGF-supplemented medium, so what they observed was the enhancement above the normal cell activity, not a “rescue” of activity, as in the case of our studies.

Importantly, biological activity of the dimers was much less dependent on the external heparin administration. In the absence of heparin, dimers performed far better than the wild-type protein in the proliferation and migration assays, as well as in the anti-apoptotic response. Although heparin and growth factors are associated with rapid and effective endothelial cell repair and in clinical studies patients with burns and diabetic foot ulcers showed decreased healing time and increased capillary circulation, heparin with its anticoagulant properties is not beneficial for patients suffering from ischemia, malnourishment, largely prolonged mitogenic and anti-apoptotic potential and vascular problems [35]. We expect that this feature is a premise for the validity of dimerization approach.

Interestingly, although we did not observe essential differences in the proliferative, anti-apoptotic or cell migration-inducing activity between investigated dimers, both FGF2 molecule arrangement and linker length were crucial in the event of FGFR1-depentent internalization. While some of dimeric variants internalized comparably to the wild-type FGF2, the C96-PEG2-C96, N-PEG12-N, N-PEG12-C, and C96-PEG12-C96 ones were characterized by significantly improved internalization yield. Hence, it seems that both mutual orientation of FGF2 molecules in the dimer and selection of the suitable linker is pivotal to obtain beneficial configuration in terms of internalization capacity.

Currently, numerous studies in the field of oncology focus on FGFRs as potential therapeutic targets, since they were found to be upregulated in many types of cancers, including lung, breast, bladder, gastric, and multiple myeloma [4,36,37,38]. In our former studies, we showed that naturally occurring FGF2, when conjugated with cytotoxic compound MMAE, can be used for specific delivery of the drug into FGFR-expressing cells [17]. Effective internalization of the bioconjugate is a key parameter in the case of delivery systems for highly cytotoxic drugs, as it allows for specific release of the active form of the cytotoxic compound only inside the target cell [39]. This makes these dimeric variants promising candidates for drug carriers in the targeted anticancer therapy.

To conclude, we designed and produced FGF2 dimers of different topology, which remained functional in terms of FGFR activation and revealed high stability, increased mitogenic potential, prolonged anti-apoptotic effect, and induced greater migration responses in normal NIH3T3 fibroblasts, as compared to FGF2 monomer. Moreover, we found out that arrangement of FGF2 molecules in dimers is critical for effective internalization into FGFR1 overexpressing cells.

## 4. Materials and Methods 

### 4.1. Antibodies and Reagents

The following primary antibodies were used: rabbit anti-p44/42 MAPK (#9102) and mouse anti-phospho-p44/42 MAPK (Thr202/Tyr204) (#9106) from Cell Signaling Technology (Danvers, MA, USA); mouse anti-γ-tubulin (#T6557) from Sigma-Aldrich (St. Louis, MO, USA); mouse anti-HSP90 (#610418) from BD Transduction Laboratory (San Jose, CA, USA). Secondary antibodies were donkey anti-mouse (#sc-2318) and donkey anti-rabbit (#sc-2077) conjugated to HRP from Santa Cruz Biotechnology (Dallas, TX, USA). Immobilon-PSQ PVDF 0.2 μm membranes were from Merck Millipore. Dulbecco’s PBS and heparin sodium salt from porcine intestinal mucosa were from Sigma-Aldrich, and Alamar Blue was from Thermo Fisher Scientific (Waltham, MA, USA). All chemical reagents were from commercial suppliers and used without further purification. Reagents used for the solid-phase peptide synthesis are as follows: TentaGel S RAM resin (particle size: 90 μm, loading 0.2–0.27 mmol/g) was from Rapp Polymere GmbH (Tübingen, Germany); Fmoc-L-Lys(Mtt)-OH, Fmoc-O2Oc-O2Oc–OH, HBTU (*O*-benzotriazole-*N,N,N′,N′*-tetramethyluroniumhexafluoro-phosphate), piperidine, TIS (triisopropylsilane), DIPEA (*N,N*-diisopropylethylamine), DMF (*N,N′*-dimethylformamide), DCM (dichloromethane), and TFA (trifluoroacetic acid) were purchased from Iris Biotech GmbH (Marktredwitz, Germany); 4-Maleimidobutyric acid was form Tokyo Chemical Industry (Tokyo, Japan); BM(PEG)2 (1,8-bismaleimido-diethyleneglycol) was form Thermo Fisher Scientific (Waltham, MA, USA); HPLC pure acetonitrile and Et_2_O (diethyl ether) were from Avantor (Gliwice, Poland). Alexa Fluor 488 NHS ester (succinimidyl ester) and DyLight 550 NHS ester were from Thermo Fisher Scientific (Waltham, MA, USA). The chromatography columns: HiTrap Desalting with Sephadex G-25 resin, HiTrap SP HP cation exchange, HiTrap Heparin HP, HiLoad 16/600 Superdex 75 pg were from GE Healthcare (Amersham, UK). Synergi 4 μm Fusion-RP 80 Å 250 × 10 mm^2^ LC column was from Phenomenex Inc. (Torrance, CA, USA). All other reagents were obtained from Sigma-Aldrich (Saint Louis, MO, USA) or BioShop Canada Inc. (Burlington, ON, Canada).

### 4.2. Cell Lines

NIH3T3 (CRL-1658) cell line was purchased from American Type Culture Collection (Manassas, VA, USA). U2OS stably transfected with FGFR1-IIIc (U2OS-R1) was a kind gift from Dr. Ellen M. Haugsten from the Department of Molecular Cell Biology, Institute for Cancer Research (Oslo University Hospital). NIH3T3 cells were grown in Dulbecco’s Modified Eagle Medium (DMEM) from Thermo Fisher Scientific, containing 10% fetal bovine serum (Thermo Fisher Scientific, Waltham, MA, USA), 100 U/mL penicillin and 100 µg/mL streptomycin (BioWest, Nuaillé, France). U2OS-R1 cells were cultivated in DMEM from BioWest supplemented with 10% fetal bovine serum and antibiotics (100 U/mL penicillin, 100 µg/mL streptomycin and 1mg/mL geneticin—BioWest). All cell lines were cultured in a humidified incubator at 37 °C in 5% CO_2_ atmosphere. Cells were seeded into tissue culture plates the day preceding the start of the experiments.

### 4.3. Protein Expression and Purification 

For the purpose of this work, three FGF2 variants differing in the position of modifiable cysteine were used: (a) FGF2 with C78S point mutation, (b) FGF2 with N-terminal KCKSGG linker and C78S C96S point mutations, and (c) FGF2 with C-terminal GGSKCK linker and C78S C96S point mutations. All proteins were produced in *Escherichia coli* Rosetta 2(DE3)pLysS expression strain from Novagen-EMD Biosciences (Madison, WI, USA), as described previously [17]. C78S point mutation was introduced via QuickChange methodology.

### 4.4. Synthesis and Purification of maleimide-PEG12-maleimide Linker

Synthesis of maleimide-PEG12-maleimide (Mal-Ado_6_-N^ε^-(Mal)-*L*-Lys-CONH_2_) was performed on solid support according to the Fmoc-SPPS approach. A mixture of TFA/DMC/TIS/H_2_O (% *v*/*v*/*v*/*v*, 95/3/1/1) was used for the final cleavage from the resin. The crude linker was triply precipitated in ice-cold Et_2_O, purified by reversed-phase high-performance liquid chromatography (HPLC), and lyophilized.

### 4.5. Synthesis of FGF2 Dimers

Step 1: Conjugation of FGF2 variants with maleimide-PEG2-maleimide and maleimide-PEG12-maleimide. Purified proteins were transferred to reaction buffer (25 mM HEPES pH 7.0, 0.5 M urea, 10 mM Na_2_SO_4_, 10 mM MgSO_4_, 5% *v*/*v* glycerin, 10 mM methionine, 1 mM EDTA, 0.05% *v*/*v* Nonidet P-40) using HiTrap desalting columns with Sephadex G-25 resin. Maleimide-PEG2-maleimide or maleimide-PEG12-maleimide linker was dissolved in *N,N*-dimethylacetamide (DMAc) at a concentration of 100 mg/mL and added directly to the protein solution (0.1 mg/mL) in a 100-fold molar excess over protein –SH groups. The conjugation reaction was carried out for 1 h at room temperature. The excess of unconjugated maleimide-PEG2-maleimide or maleimide-PEG12-maleimide was removed by ion-exchange chromatography using an SP Sepharose column. PEGylated FGF2 was eluted from the column with 25 mM HEPES pH 7.0, 0.5 M urea, 10 mM Na_2_SO_4_, 10 mM MgSO_4_, 5% *v*/*v* glycerin, 1 mM EDTA, 0.5 M NaCl. 

Step 2: Dimerization Reaction. For the reaction, both PEGylated and unPEGylated FGF2 variants were used at the highest possible concentration (~1 mg/mL). UnPEGylated FGF2 rebuffered to the reaction buffer was added directly to the PEGylated FGF2 eluted from the SP Sepharose column, to give 1.5–2.5 molar excess. The reaction mixture was incubated with stirring at 15 °C for 12 h. Finally, proteins were concentrated on the HeparinSepharose column, and subsequently, dimer was separated via size-exclusion chromatography using HiLoadSuperdex 75 pg column.

### 4.6. Spectrofluorimetry

Spectrofluorimetric analysis was performed to determine the folding state of dimers. Tryptophan fluorescence spectra were acquired using a FP-750 spectrofluorometer (Jasco, Japan) with excitation at 280 nm and emission in the 300–450 nm range. The concentration of protein was ~2 × 10^−6^ M in Dulbecco’s PBS.

### 4.7. Mass Spectrometry

The identity of dimers was confirmed by MALDI-TOF/TOF MS (Applied Biosystem AB 4800+). α-cyano-4-hydroxycinnamic acid or sinapic acid served as a matrix.

### 4.8. Activation of Signaling Pathways

NIH3T3 and U2OS-R1 cells were serum-deprived, starved for 8 h, and stimulated for 15 min at 37 °C with 10 ng/mL FGF2 WT or dimers. The cells were then washed with PBS, lysed with Laemmli Sample Buffer, and sonicated. Total cell lysates were separated by SDS-PAGE and subjected to western blot analysis with the anti-phospho-p44/42 (Thr202/Tyr204) MAP kinase antibody (p-ERK1/2) and anti-p44/42 MAP kinase antibody (ERK1/2). Anti-γ-tubulin or anti-Heat Shock Protein 90 (HSP90) antibody was used as a loading control. All primary antibodies were used at the 1/10000 dilution. Appropriate secondary antibodies conjugated to HRP and an enhanced chemiluminescent substrate were used for specific protein band visualization in a ChemiDoc station (BioRad, Hercules, CA, USA).

### 4.9. Protein Stability in Cell-Conditioned Medium

NIH3T3 cells were seeded onto 12-well plates (100,000 cells/well), serum-starved, and then, supplemented with 10 ng/mL FGF2 WT or dimers. After 24 and 48 h of incubation at 37 °C, conditioned media were collected, centrifuged to remove cell debris and transferred to the new set of serum-starved NIH3T3 cells. Activation of cell signaling cascades was used as an evidence of FGF2 monomer and dimers’ degradation progress. Freshly prepared 10 ng/mL FGF2 solution served as a positive control. Cells were stimulated for 15 min at 37 °C and lysed with Laemmli Sample Buffer. Total cell lysates were separated by SDS-PAGE and analyzed by western blotting, as described above.

### 4.10. Cell Proliferative Activity Assay

NIH3T3 cells grown on 96-well culture plates (10,000 cells/well) were serum-starved for 24 h, and then, treated with increasing concentrations (1, 3, 10, and 30 ng/mL) of the samples in the presence or absence of heparin (10 U/mL). After 72 h of incubation without media exchange, cell proliferative potential was assessed by addition of AlamarBlue Cell Viability Reagent in a concentration of 10% *v*/*v*, directly to the culture medium. After 4 h of incubation, the emission of fluorescent reduced form of the dye was measured at 590 nm upon excitation at 560 nm on an Infinite M1000 PRO plate reader (Tecan, Männedorf, Switzerland). All experimental groups were normalized to the control group, which received only blank medium.

### 4.11. Cell Counting

Total cell counting was performed using the trypan blue (Thermo Fisher Scientific, Waltham, MA, USA) dye exclusion method and the TC20 Automated Cell Counter (BioRad, Hercules, CA, USA).

### 4.12. Detection of Apoptosis

The apoptosis study was carried out by flow cytometry using NovoCyte 2060R Flow Cytometer (ACEA Biosciences, San Diego, CA, USA) and the Annexin V Apoptosis Detection Kit FITC (Thermo Fisher Scientific, Waltham, MA, USA). For the analysis, 100,000 NIH3T3 cells were seeded onto each well of a 12-well culture plate and allowed to adhere overnight. The cells were subsequently starved for 8 h with serum-free medium, and then, treated with 10 ng/mL FGF2 WT or dimers in the presence or absence of 10 U/mL heparin. After 72 h of incubation, the cells were harvested with TrypLE Express solution (Thermo Fisher Scientific), pelleted and rinsed extensively with PBS. Annexin V-FITC and propidium iodide staining was performed according to the manufacturer’s protocol. Excessive dyes were washed off with PBS and samples were subjected to FACS analysis. The data were analyzed with NovoExpress Software (ACEA Biosciences, San Diego, CA, USA).

### 4.13. Cell Migration Assay

Cell movement into wound region was imaged and measured in standard scratch wound assay with the use of an IncuCyte® Cell Migration and Invasion System (Essen BioScience, Ann Arbor, MI, USA). NIH3T3 cells were seeded onto an IncuCyte® ImageLock 96-well plate and serum-starved for 24 h. Cell-free zones in cell monolayers were created with the IncuCyte® WoundMaker tool. The cells were stimulated with 10 ng/mL FGF2 or dimers in the presence or absence of heparin (10 U/mL) for 48 h. The images were acquired automatically every 2 h. The data were analyzed with the support of IncuCyte® ZOOM Software.

### 4.14. Protein Labeling with Fluorescent Probe 

Unmodified FGF2 WT and all FGF2 dimers were labeled with Alexa Fluor 488 NHS Ester. An amount of 30 µL of the fluorescent dye reconstituted in DMAC (1 mg/mL) was added directly to 600 µL of purified proteins (FGF2 WT or dimers) at a concentration of 70 µg/mL in PBS adjusted to pH 8.0. The incubation was maintained at room temperature in the dark for 1 h. Unreacted dye was removed with the use of mini spin columns packed with heparin-Sepharose affinity resin. Moreover, for microscopic analysis of the internalization, FGF2 WT and C96-PEG2-C96, N-PEG12-N, N-PEG12-C, C96-PEG12-C96 dimers were labeled with DyLight 550 NHS Ester. An amount of 2.5 µL of fluorescent probe (10 mg/mL) was added to 100 µL of protein sample at 0.5 mg/mL concentration in 0.05 M sodium borate buffer at pH 8.0. The reaction was carried out at room temperature for 1 h in the dark. Unreacted dye was removed using Zeba Spin Desalting columns (Thermo Fisher Scientific, Waltham, MA, USA).

### 4.15. Flow Cytometric Analysis of Steady-State Internalization

Method for flow cytometric assessment of ligand internalization efficiency was adapted from the literature [40]. To determine the internalization yield depending on the concentration of the ligand, serum-starved U2OS-R1 cells were incubated with increasing concentrations of Alexa Fluor 488-labeled proteins (3, 10, and 30 ng/mL) in the presence of 10 U/mL heparin at 37 °C for 20 min. In turn, for the study of internalization kinetics, 100 ng/mL of Alexa Fluor 488-labeled proteins was added to serum-starved cells in the presence of heparin and the incubation was carried out for 5, 10, 15, or 30 min at 37 °C. In order to stop receptor trafficking, cells were rapidly placed on ice and washed with ice-cold PBS. Non-internalized ligand was removed from the cell surface by multiple washes in acid stripping buffer. The cells were detached from the culture plate with 5 mM EDTA-PBS, washed in FACS buffer, and subjected for flow cytometric analysis using a NovoCyte 2060R instrument and NovoExpress software (ACEA Biosciences, San Diego, CA, USA).

### 4.16. Bio-Layer Interferometry (BLI) Analysis of Binding of Alexa Fluor 488-Labeled C-PEG12-C Dimer to FGFR1c

Binding of Alexa Fluor 488-labeled C-PEG12-C dimer to the FGFR1c was confirmed using ForteBio Octet K2 (Pall ForteBio, Fremont, CA, USA) and Streptavidin biosensors (SA) (Pall ForteBio, Fremont, CA, USA). Analysis of affinity of Alexa Fluor 488-labeled and non-labeled (control) C-PEG12-C dimer, to extracellular domains of FGFR1c fused to Fc fragments was performed at 20 °C in PBS with 0.2% (*w*/*v*) BSA, 0.1% (*w*/*v*) PEG 3.5 kDa, 0.05% (*v*/*v*) Triton X-100, and 10 mM (NH_4_)_2_SO_4_. The wells in the 96-well black plate were filled with 200 μL of sample and incubated for 20 min at 20 °C for system equilibration. Then, biotinylated FGFR1c was immobilized on the SA sensor for 300 s, the sensor was blocked with biocytin (0.04 mg/mL) for 30 s and washed for 60 s. Association of the Alexa Fluor 488-labeled and non-labeled C-PEG12-C dimer at 20 nM concentration was carried out for 200 s, and the dissociation was monitored for another 200 s. A simple 1:1 Langmuir model was used for fitting using Octet Data Analysis 11.0 software.

### 4.17. Evaluation of Biological Competence of AF488-Labeled C-PEG12-C Dimer

NIH3T3 and U2OS-R1 cells were serum-starved for 8 h, and then, stimulated for 15 min at 37 °C with 30 ng/mL of Alexa Fluor 488-labeled C-PEG12-C dimer. Activation of ERKs cascades was used as an evidence of dimers’ biological activity. FGF2 WT and non-labeled C-PEG12-C dimer used at the same concentration served as a positive control. Total cell lysates were separated by SDS-PAGE and analyzed by western blotting, as described in paragraph 4.8.

### 4.18. Fluorescence Microscopy: Internalization of FGF2 Dimers into Cells Overproducing FGFR1

For microscopic examination of the internalization of selected dimers, serum-starved U2OS-R1 cells grown on a 96-well glass bottom plate were pre-incubated with 100 ng/mL of DyLight 550-labeled wild-type FGF2 or dimeric variants in serum-free medium supplemented with 1% BSA and 10 U/mL heparin for 10 min on ice. Then, the cells were transferred to 37 °C and incubation was continued for another 15 min. The cells were subsequently washed with PBS, fixed in 4% formaldehyde solution and blocked with 1% BSA and 0.1 M glycine in PBS. Nuclei were counterstained with DAPI. Wide-field fluorescence microscopy was carried out using a Zeiss Axio Observer Z1 fluorescence microscope. Images were taken with a LD-Plan-Neofluar 63×/0.75 Corr M27 objective and Axiocam 503 camera (Zeiss, Oberkochen, Germany). The fluorescence of DyLight 550 was visualized with a 540/522 nm bandpass excitation filter and a 575/640 nm bandpass emission filter. DAPI signal was visualized with a 335/383 nm bandpass excitation filter and a 420/470 nm emission filter. Image analysis was carried out with Zeiss ZEN 2.6 software (Zeiss, Oberkochen, Germany) and Adobe Illustrator CC (Adobe, San Jose, CA, USA).

### 4.19. Statistical Analysis

The data were analyzed by one-way analysis of variance (ANOVA) followed by Tukey’s post hoc multiple comparison test using GraphPadPrism 6 software (San Diego, CA, USA). Statistical significance was determined versus FGF2 WT group. A p-value of less than 0.05 was considered statistically significant.

## Supplementary Materials

Supplementary materials can be found at https://www.mdpi.com/1422-0067/21/11/4108/s1.

## Abbreviations

FGF	fibroblast growth factor
FGFR	fibroblast growth factor receptor
RTK	receptor tyrosine kinase
HSPG	heparan sulfate proteoglycans
SEC	size exclusion chromatography
IEC	ion exchange chromatography
PEG	poly(ethylene glycol)
Ado	8-amino-3,6-dioxaoctanoyl
MMAE	monomethyl auristatin E

## References

[1] Yun Y., Won J.E., Jeon E., Lee S., Kang W., Jo H., Jang J.-H., Shin U.S., Kim H. (2010). Fibroblast Growth Factors: Biology, Function, and Application for Tissue Regeneration. J. Tissue Eng..

[2] Beenken A., Mohammadi M. (2009). The FGF family: Biology, pathophysiology and therapy. Nat. Rev. Drug Discov..

[3] Ornitz D.M., Itoh N. (2015). The Fibroblast Growth Factor signaling pathway. Wiley Interdiscip. Rev. Dev. Biol..

[4] Turner N., Grose R. (2010). Fibroblast growth factor signalling: From development to cancer. Nat. Rev. Cancer.

[5] Eswarakumar V.P., Lax I., Schlessinger J. (2005). Cellular signaling by fibroblast growth factor receptors. Cytokine Growth Factor Rev..

[6] Plotnikov A.N., Schlessinger J., Hubbard S.R., Mohammadi M. (1999). Structural Basis for FGF Receptor Dimerization and Activation. Cell.

[7] Schlessinger J., Plotnikov A.N., Ibrahimi O.A., Eliseenkova A.V., Yeh B.K., Yayon A., Linhardt R.J., Mohammadi M. (2000). Crystal Structure of a Ternary FGF-FGFR-Heparin Complex Reveals a Dual Role for Heparin in FGFR Binding and Dimerization. Mol. Cell.

[8] Marianayagam N.J., Sunde M., Matthews J.M. (2004). The power of two: Protein dimerization in biology. Trends Biochem. Sci..

[9] Kramer R.H., Karpen J.W. (1998). Spanning binding sites on allosteric proteins with polymer-linked ligand dimers. Nature.

[10] Safran M., Eisenstein M., Aviezer D., Yayon A. (2000). Oligomerization reduces heparin affinity but enhances receptor binding of fibroblast growth factor 2. Biochem. J..

[11] Kwan C., Venkataraman G., Shriver Z., Raman R., Liu D., Qi Y., Varticovski L., Sasisekharan R. (2001). Probing Fibroblast Growth Factor Dimerization and Role of Heparin-like Glycosaminoglycans in Modulating Dimerization and Signaling. J. Biol. Chem..

[12] Decker C.G., Wang Y., Paluck S.J., Shen L., Loo J.A., Levine A.J., Miller L.S., Maynard H.D. (2016). Fibroblast growth factor 2 dimer with superagonist in vitro activity improves granulation tissue formation during wound healing. Biomaterials.

[13] Stauber D.J., DiGabriele A.D., Hendrickson W.A. (2000). Structural interactions of fibroblast growth factor receptor with its ligands. Proc. Natl. Acad. Sci. USA.

[14] Herr A.B., Ornitz D.M., Sasisekharan R., Venkataraman G., Waksman G. (1997). Heparin-induced self-association of fibroblast growth factor-2. Evidence for two oligomerization processes. J. Biol. Chem..

[15] Venkataraman G., Sasisekharan V., Herr A.B., Ornitz D.M., Waksman G., Cooney C.L., Langer R., Sasisekharan R. (1996). Preferential self-association of basic fibroblast growth factor is stabilized by heparin during receptor dimerization and activation. Proc. Natl. Acad. Sci. USA.

[16] Davis J.C., Venkataraman G., Shriver Z., Raj P.A., Sasisekharan R. (1999). Oligomeric self-association of basic fibroblast growth factor in the absence of heparin-like glycosaminoglycans. Biochem. J..

[17] Krzyscik M.A., Zakrzewska M., Sørensen V., Sokolowska-Wedzina A., Lobocki M., Swiderska K.W., Krowarsch D., Wiedlocha A., Otlewski J. (2017). Cytotoxic Conjugates of Fibroblast Growth Factor 2 (FGF2) with Monomethyl Auristatin E for Effective Killing of Cells Expressing FGF Receptors. ACS Omega.

[18] Lobocki M., Zakrzewska M., Szlachcic A., Krzyscik M.A., Sokolowska-Wedzina A., Otlewski J. (2017). High-Yield Site-Specific Conjugation of Fibroblast Growth Factor 1 with Monomethylauristatin E via Cysteine Flanked by Basic Residues. Bioconjug. Chem..

[19] Boilly B., Vercoutter-Edouart A.S., Hondermarck H., Nurcombe V., Le Bourhis X. (2000). FGF signals for cell proliferation and migration through different pathways. Cytokine Growth Factor Rev..

[20] LaVallee T.M., Prudovsky I.A., McMahon G.A., Hu X., Maciag T. (1998). Activation of the MAP Kinase Pathway by FGF-1 Correlates with Cell Proliferation Induction While Activation of the Src Pathway Correlates with Migration. J. Cell Biol..

[21] Szlachcic A., Sochacka M., Czyrek A., Opalinski L., Krowarsch D., Otlewski J., Zakrzewska M. (2019). Low Stability of Integrin-Binding Deficient Mutant of FGF1 Restricts Its Biological Activity. Cells.

[22] Bruno M., Rizzo I.M., Romero-Guevara R., Bernacchioni C., Cencetti F., Donati C., Bruni P. (2017). Sphingosine 1-phosphate signaling axis mediates fibroblast growth factor 2-induced proliferation and survival of murine auditory neuroblasts. Biochim. Biophys. Acta Mol. Cell Res..

[23] Zhao Y., Cao F., Yu X., Chen C., Meng J., Zhong R., Zhang Y., Zhu D. (2018). Linc-RAM is required for FGF2 function in regulating myogenic cell differentiation. RNA Biol..

[24] Awan B., Turkov D., Schumacher C., Jacobo A., McEnerney A., Ramsey A., Xu G., Park D., Kalomoiris S., Yao W. (2018). FGF2 Induces Migration of Human Bone Marrow Stromal Cells by Increasing Core Fucosylations on N-Glycans of Integrins. Stem Cell Rep..

[25] Nunes Q.M., Li Y., Sun C., Kinnunen T.K., Fernig D.G. (2016). Fibroblast growth factors as tissue repair and regeneration therapeutics. PeerJ.

[26] Akita S., Akino K., Imaizumi T., Hirano A. (2008). Basic fibroblast growth factor accelerates and improves second-degree burn wound healing. Wound Repair Regen..

[27] Matsumine H. (2015). Treatment of Skin Avulsion Injuries with Basic Fibroblast Growth Factor. Plast. Reconstr. Surg. Glob. Open.

[28] Kawaguchi H., Oka H., Jingushi S., Izumi T., Fukunaga M., Sato K., Matsushita T., Nakamura K. (2010). A local application of recombinant human fibroblast growth factor 2 for tibial shaft fractures: A randomized, placebo-controlled trial. J. Bone Miner. Res..

[29] Kitamura M., Akamatsu M., Machigashira M., Hara Y., Sakagami R., Hirofuji T., Hamachi T., Maeda K., Yokota M., Kido J. (2011). FGF-2 Stimulates Periodontal Regeneration. J. Dent. Res..

[30] Uchi H., Igarashi A., Urabe K., Koga T., Nakayama J., Kawamori R., Tamaki K., Hirakata H., Ohura T., Furue M. (2009). Clinical efficacy of basic fibroblast growth factor (bFGF) for diabetic ulcer. Eur. J. Dermatol..

[31] Kumagai M., Marui A., Tabata Y., Takeda T., Yamamoto M., Yonezawa A., Tanaka S., Yanagi S., Ito-Ihara T., Ikeda T. (2016). Safety and efficacy of sustained release of basic fibroblast growth factor using gelatin hydrogel in patients with critical limb ischemia. Heart Vessel..

[32] Carter E.P., Fearon A.E., Grose R.P. (2015). Careless talk costs lives: Fibroblast growth factor receptor signalling and the consequences of pathway malfunction. Trends Cell Biol..

[33] Estapé D., van denHeuvel J., Rinas U. (1998). Susceptibility towards intramolecular disulphide-bond formation affects conformational stability and folding of human basic fibroblast growth factor. Biochem. J..

[34] Sanz J.M., Gimenez-Gallego G. (1997). A partly Folded State of Acidic Fibroblast Growth Factor at Low Ph. Eur. J. Biochem..

[35] Galvan L. (1996). Effects of heparin on wound healing*1. J. WOCN.

[36] Wesche J., Haglund K., Haugsten E.M. (2011). Fibroblast growth factors and their receptors in cancer. Biochem. J..

[37] Katoh M. (2016). FGFR inhibitors: Effects on cancer cells, tumor microenvironment and whole-body homeostasis (Review). Int. J. Mol. Med..

[38] Schmitz K., Schildhaus H.-U. (2014). Clinical significance of FGFR1 gene amplification in lung cancer patients. Lung Cancer Manag..

[39] Ducry L., Stump B. (2010). Antibody–Drug Conjugates: Linking Cytotoxic Payloads to Monoclonal Antibodies. Bioconjug. Chem..

[40] Li N., Hill K.S., Elferink L.A. (2008). Analysis of Receptor Tyrosine Kinase Internalization Using Flow Cytometry. Membrane Trafficking.

