# Supplementary material for: Stable Fibroblast Growth Factor 2 Dimers with High Pro-Survival and Mitogenic Potential
Source: Int J Mol Sci. 2020 Jun 9;21(11):4108. doi: 10.3390/ijms21114108 (PMC7312490; doi:10.3390/ijms21114108)
Supplement: Supplementary file 1 [file ijms-21-04108-s001.pdf]

International Journal of *Molecular Sciences*

MDPI

Supplementary Materials

Stable Fibroblast Growth Factor 2 Dimers with High Pro-Survival and Mitogenic Potential

Daria Nawrocka [1], Mateusz Adam Krzyscik [1,2], Łukasz Opaliński [1], Malgorzata Zakrzewska [1] and Jacek Otlewski [1,*]

1 Department of Protein Engineering, Faculty of Biotechnology, University of Wroclaw, Joliot-Curie 14a, 50-383 Wroclaw, Poland; daria.nawrocka@uwr.edu.pl (D.N.); mateusz.krzyscik@uwr.edu.pl (M.A.K.); lukasz.opalinski@uwr.edu.pl (Ł.O.); malgorzata.zakrzewska@uwr.edu.pl (M.Z.)

2 Department of Protein Biotechnology, Faculty of Biotechnology, University of Wroclaw, Joliot-Curie 14a, 50-383 Wroclaw, Poland

* Correspondence: jacek.otlewski@uwr.edu.pl; Tel.: +48-71-375-28-24

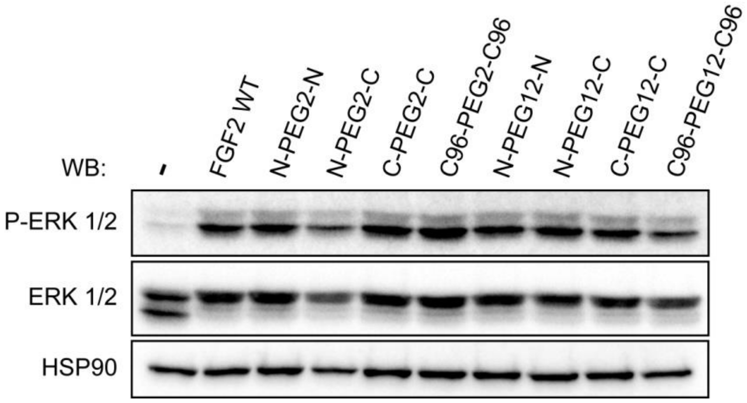

Figure S1. Activation of cellular signaling. Serum-starved U2OS-R1 cells were stimulated for 15 minutes with wild-type FGF2 (FGF2 WT) or dimers at 10 ng/mL concentration. Cells were lysed and activation of downstream signaling cascades was assessed with western blotting using the following antibodies: anti-phospho-ERK1/2 (P-ERK1/2), anti-ERK1/2 and anti-heat shock protein 90 (HSP90) as a loading control.

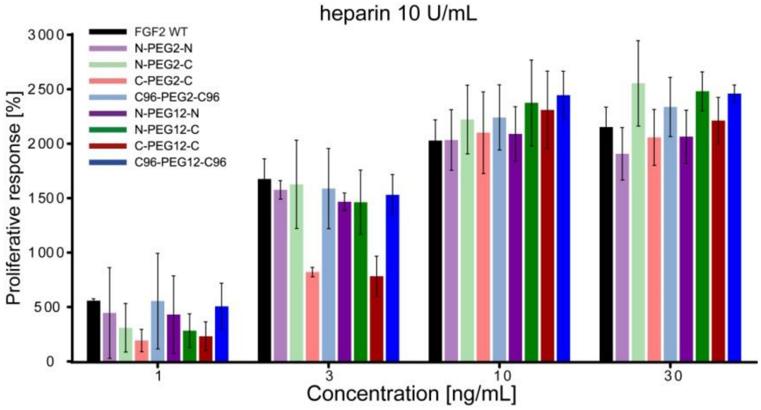

Figure S2. Biological activity of FGF2 dimers. Serum-starved NIH3T3 cells were treated with wild-type FGF2 (FGF2 WT) or dimers at various concentrations (1-30 ng/mL) in the presence of 10 U/mL heparin. After 72 h cell viability was measured using AlamarBlue Reagent. Percent

proliferative activity of NIH3T3 (mean ± SD) was normalized to the blank media per treatment set. The average values and errors were calculated based on three independent experiments.

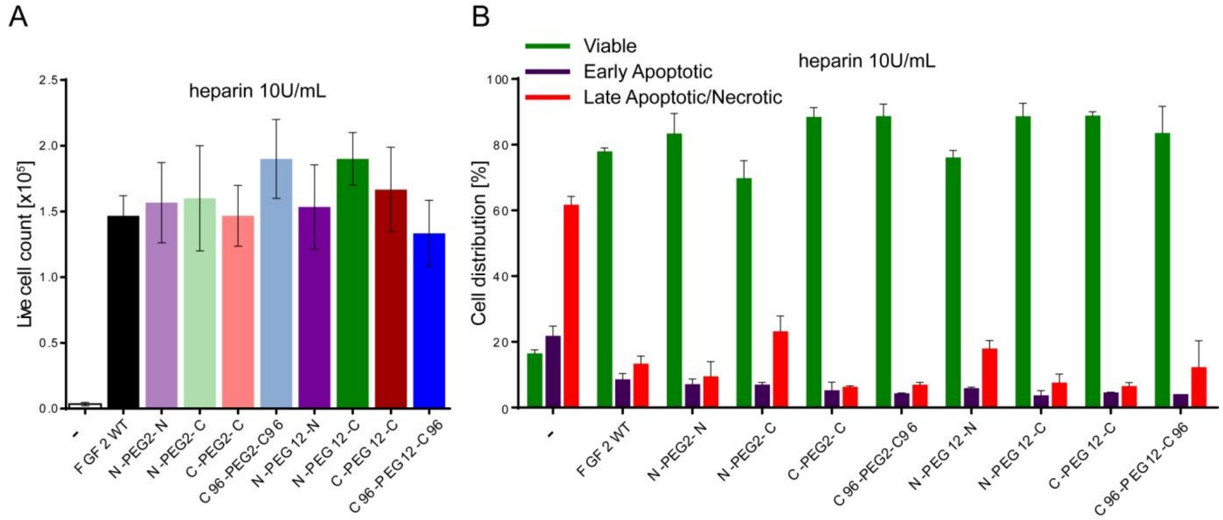

Figure S3. Pro-survival effect of FGF2 dimers on NIH3T3 cells. (**A**) Live cell counting performed with the use of hemocytometer and Trypan Blue staining after 72 h of culture with 10 ng/mL wild-type FGF2 (FGF2 WT) or dimers in the presence of 10 U/mL heparin. (**B**) Apoptosis assessed by Annexin V and propidium iodide (PI) assay. The data are mean values of three independent experiments ± SD.

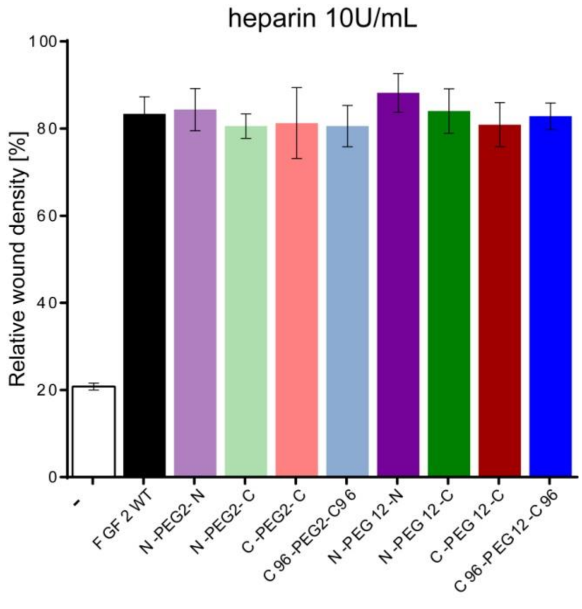

Figure S4. The effect of dimeric FGF2 variants on the migration of NIH3T3 cells. Serum-starved NIH3T3 cells were treated with FGF2 dimers (10 ng/mL) in the presence of 10 U/mL heparin. Relative wound density was calculated after 48 h of cell stimulation. Representative results of one of two independent experiments are shown. The mean values ± SD were calculated based on four replicates.

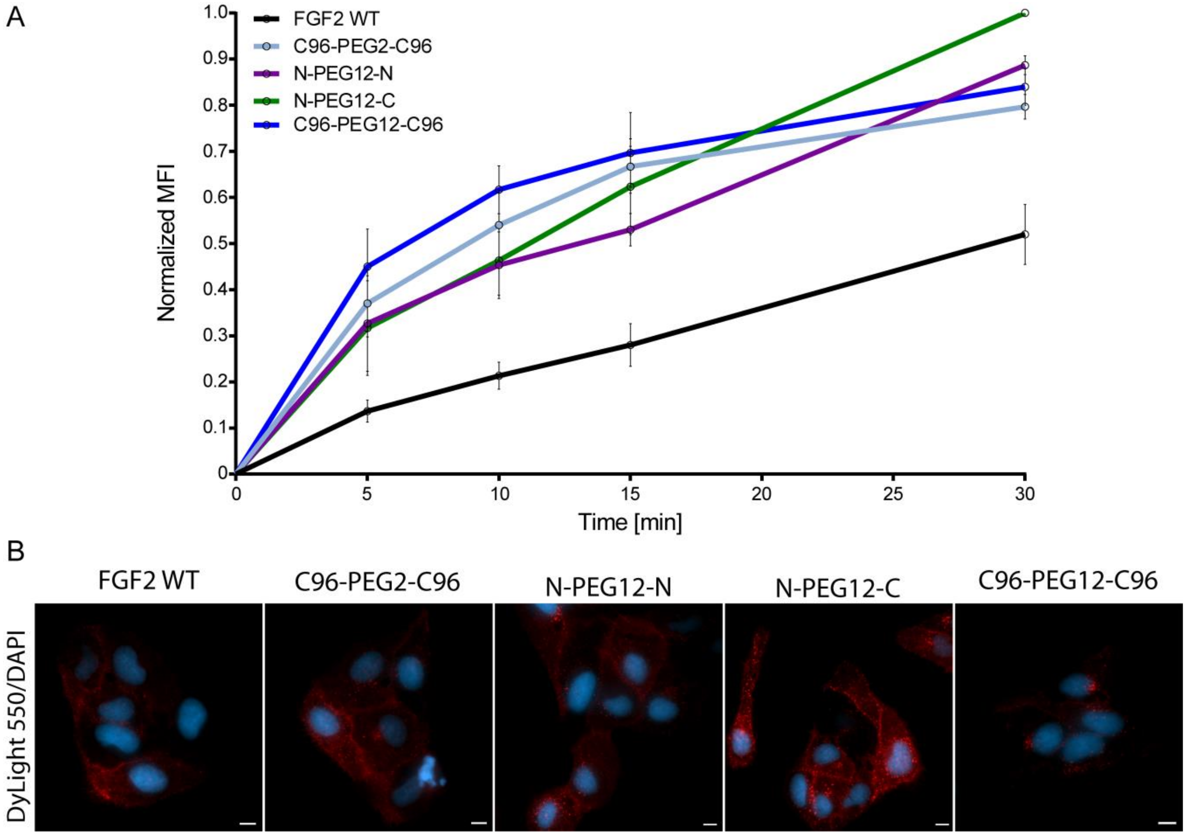

Figure S5. Internalization of FGF2 dimers. (**A**) Flow cytometric study of internalization kinetics. 100 ng/mL of Alexa Fluor 488-labeled proteins was added to cells, and the incubation was carried out for 5, 10, 15, or 30 minutes at 37 °C. Results represent the mean fluorescence intensities (MFI) normalized to untreated control cells under each experimental condition from three independent experiments. Values are the means for each data set ± SD. (**B)** Serum-starved U2OS-R1 cells were pre-incubated with DyLight 550-labeled wild-type FGF2 or selected dimers (100 ng/mL) for 10 minutes on ice and then transferred to 37 ˚C for 15 minutes. Cells were subsequently fixed and nuclei were visualized with DAPI. Analysis was performed by fluorescence microscopy. Scale bars represent 10 μm.

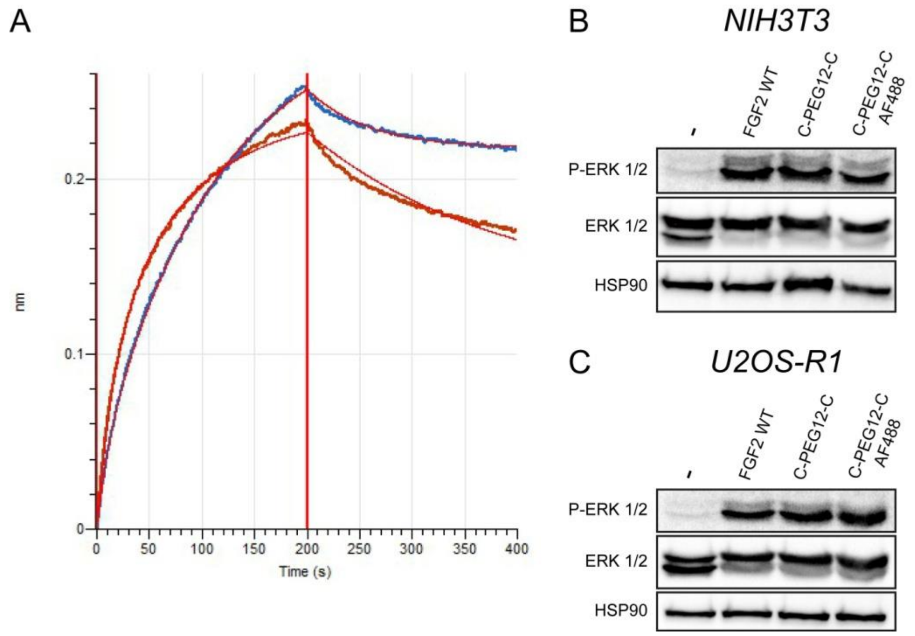

Figure S6. Binding properties and biological functionality of Alexa Fluor 488 (AF488)-labeled C-PEG12-C dimer. (**A**) Bio-layer interferometry (BLI) analysis of the interaction of AF-488-labeled and non-labeled C-PEG12-C dimer with FGFR1c. The solid lines represent local fits to the 1:1 interaction model. (**B**,**C**) Activation of ERKs cascades by AF-488 labeled and non-labeled C-PEG12-C dimer in NIH3T3 (**B**) and U2OS-R1 (**C**) cells assessed with western blotting.

© 2020 by the authors. Submitted for possible open access publication under the terms and conditions of the Creative Commons Attribution (CC BY) license (http://creativecommons.org/licenses/by/4.0/).
